# Linking Infectious and Narcology Care (LINC) in Russia: design, intervention and implementation protocol

**DOI:** 10.1186/s13722-016-0058-5

**Published:** 2016-05-04

**Authors:** Natalia Gnatienko, Steve C. Han, Evgeny Krupitsky, Elena Blokhina, Carly Bridden, Christine E. Chaisson, Debbie M. Cheng, Alexander Y. Walley, Anita Raj, Jeffrey H. Samet

**Affiliations:** Clinical Addiction Research and Education (CARE) Unit, Department of Medicine, Section of General Internal Medicine, Boston Medical Center, 801 Massachusetts Avenue, 2nd Floor, Boston, MA 02118 USA; Clinical Addiction Research and Education (CARE) Unit, Department of Medicine, Section of General Internal Medicine, Boston University School of Medicine/Boston Medical Center, 801 Massachusetts Avenue, 2nd Floor, Boston, MA 02118 USA; First St. Petersburg Pavlov State Medical University, Lev Tolstoy St., 6/8, St. Petersburg, Russian Federation 197022; St. Petersburg Bekhterev Research Psychoneurological Institute, Bekhtereva St., 3, St. Petersburg, Russian Federation 192019; Data Coordinating Center, Boston University School of Public Health, 85 East Newton Street, 9th Floor, Boston, MA 02118 USA; Department of Biostatistics, Boston University School of Public Health, 801 Massachusetts Avenue, 3rd Floor, Boston, MA 02118 USA; Department of Medicine, Division of Global Public Health, University of California-San Diego, 9500 Gilman Dr. MC 0507, San Diego, CA 92093 USA; Department of Community Health Sciences, Boston University School of Public Health, 801 Massachusetts Avenue, 2nd Floor, Boston, MA 02118 USA

**Keywords:** HIV treatment, Substance use, Russian HIV, Peer case managers

## Abstract

**Background:**

Russia and Eastern Europe have one of the fastest growing HIV epidemics in the world. While countries in this region have implemented HIV testing within addiction treatment systems, linkage to HIV care from these settings is not yet standard practice. The Linking Infectious and Narcology Care (LINC) intervention utilized peer-led strengths-based case management to motivate HIV-infected patients in addiction treatment to obtain HIV care. This paper describes the protocol of a randomized controlled trial evaluating the effectiveness of the LINC intervention in St. Petersburg, Russia.

**Methods/design:**

Participants (n = 349) were recruited from the inpatient wards at the City Addiction Hospital in St. Petersburg, Russia. After completing a baseline assessment, participants were randomly assigned to receive either the LINC intervention or standard of care. Participants returned for research assessments 6 and 12 months post-baseline. Primary outcomes were assessed via chart review at HIV treatment locations.

**Discussion:**

LINC holds the potential to offer an effective approach to coordinating HIV care for people who inject drugs in Russia. The LINC intervention utilizes existing systems of care in Russia, minimizing adoption of substantial infrastructure for implementation.

*Trial Registration* NCT01612455

## Background

The HIV epidemic in Russia and Eastern Europe is one of the most rapidly expanding HIV epidemics in the world, with transmission risk primarily from injection drug use [1–7]. The total number of people living with HIV in Russia is estimated at 850,000–1,300,000 [8]. The strategy to seek, test, treat and retain (STTR) is a useful paradigm to facilitate progress along the HIV cascade from identification (i.e., HIV testing) to treatment and viral suppression [9, 10]. Russia and other countries in the region were early adopters of routine HIV testing within the established addiction treatment systems (e.g., narcology hospitals as referred to in Russia), but have failed to effectively link HIV-infected patients to HIV care [3, 4, 11]. This represents a missed opportunity. It is estimated that 80 % of those infected with HIV in Russia are people who inject drugs (PWID) and up to 33 % of Russian PWID receiving narcology treatment are HIV-infected [6, 12]. Yet, PWID account for only 20–30 % of patients receiving antiretroviral therapy (ART) [6, 13]. As was the case in the United States in the 1990s, delayed or non-receipt of HIV medical care, particularly among PWID, is common in Russia, even though many are aware of their diagnosis [9, 11, 14–17]. Recently, the Russian AIDS treatment system began using case managers (CMs) to help retain patients in HIV care [18]. Unfortunately, these efforts have not been focused on care initiation, nor are they being operationalized in narcology treatment settings. In addition, existing CMs do not address the unique challenges faced by Russian PWID that impact their retention in HIV care, such as stigma and the competing priorities for those with addiction [19–21]. Helping PWID face the challenges of stigma and their addiction is vital given the difficulty of initiating and retaining PWID in HIV care [17].

To address this recognized gap in HIV care in Russia, as well as elsewhere in the world, we sought to test an integrated behavioral and structural intervention (i.e., an intervention addressing behavioral risks and structurally linking substance abuse treatment and HIV care) to improve the treat and retain dimensions of the STTR paradigm. This intervention, Linking Infectious and Narcology Care (LINC), involved peer-led strengths-based case management to support and motivate HIV-infected PWID in a narcology hospital in St. Petersburg, Russia to link with HIV medical care, thus facilitating coordinated care between the narcology and HIV medical systems [22]. Here we describe study design, intervention and control procedures, and implementation of the protocol.

## Study methods/design

We conducted a randomized controlled trial to assess the effectiveness of the LINC intervention among HIV-infected Russian PWID compared to standard of care treatment in Russia on the following outcomes: (1) initiation of HIV care (i.e., 1 or more visit to HIV medical care) within 6 months of enrollment; (2) retention in HIV care (i.e., 1 or more visit to HIV medical care in two consecutive 6 month periods) within 12 months of enrollment; (3) appropriate HIV care (i.e., prescribed ART if CD4 cell count was <350 or having a second CD4 count if CD4 > 350) within 12 months of enrollment; and (4) improved HIV health outcomes (i.e., CD4 cell count) 12 months after enrollment.

### Study setting

Participants were recruited from inpatient wards at the City Addiction Hospital in St. Petersburg, Russia from July 2012 through May 2014. The St. Petersburg City Addiction Hospital is a government-funded 500-bed hospital, providing free addiction care to residents of the city of St. Petersburg, who are registered as having a substance use disorder (drug or alcohol). The hospital provides detoxification, early stabilization, including the treatment of co-morbid psychiatric and somatic disorders, and inpatient rehabilitation. Typical length of stay for hospitalized patients is one to 3 weeks.

### Participants and recruitment

The study enrolled 349 participants who met the following inclusion criteria: (1) age 18–70 years; (2) HIV-infected; (3) hospitalized at the narcology hospital; (4) history of injection drug use; (5) agree to CD4 cell count testing; (6) have two contacts to assist with follow-up; (7) live within 100 km of St. Petersburg; (8) have a telephone; (9) willing to receive HIV care at Botkin Infectious Disease Hospital. The following served as exclusion criteria for study enrollment: (1) currently on ART; (2) not fluent in Russian; (3) cognitive impairment precluding informed consent.

All HIV-infected patients admitted to the intensive care and detoxification/rehabilitation departments of the City Addiction Hospital were eligible for screening. At the City Addiction Hospital, information about a patient’s HIV status is routinely collected in the admission department and noted on the front page of the patient’s medical chart. Three times a week, a nurse identified medical charts of HIV-infected patients who had not been previously screened. Patients were screened 1–5 days after admission to the narcology hospital and after treatment for most severe initial withdrawal symptoms. Screening was conducted by Research Assessors (RAs) who were City Addiction Hospital physicians with narcology subspecialty training (i.e., narcologists). They had access to medical charts and were trained on the research protocol by the Russian and US study investigators (EB, CC). Narcologists who were not involved with the patient’s medical care approached the patient to assess study eligibility. The RA initiated pre-screening procedures of potentially appropriate patients by reviewing the medical chart to assess exclusion criteria (e.g., significant cognitive impairment, currently on ART).

**Fig. 1:**
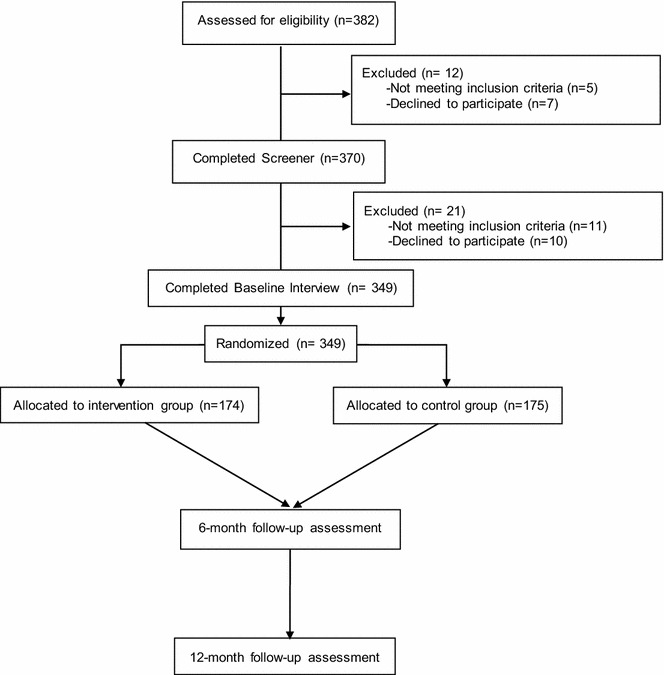
CONSORT diagram

Once a patient was identified as being HIV-infected based on chart review, but not having evident significant cognitive impairment, not on ART, and not previously approached about the LINC study, the RA met with the patient in a private location (e.g., hospital room or exam room) to briefly describe the study and conduct the screening to confirm the presence of inclusion criteria and absence of exclusion criteria. The RA offered eligible participants enrollment into the study and as appropriate, administered and documented the informed consent. Out of 382 participants, whose charts were reviewed as part of the pre-screening process, 370 (96.9 %) were eligible and agreed to be screened. Out of those, 359 (97.0 %) met eligibility criteria to participate in the study. The main reason for ineligibility was having taken ART in the past 30 days (Table 1). Out of those who were eligible, 349 (97.2 %) were enrolled and randomized (Fig. 1).

**Table 1:** Reasons for ineligibility for study participation

	n
Total ineligible during pre-screening	
HIV diagnosis not confirmed	4
Significant cognitive impairment	1
Total ineligible on screener	
Taken ART in past 30 days	4
Not enrolled due to RA discretion	3
Never injected drugs	1
Not available for CD4 testing	1
Not willing to receive care at Botkin	1
Does not have two contacts to assist with follow-up	1

**Fig. 2:**
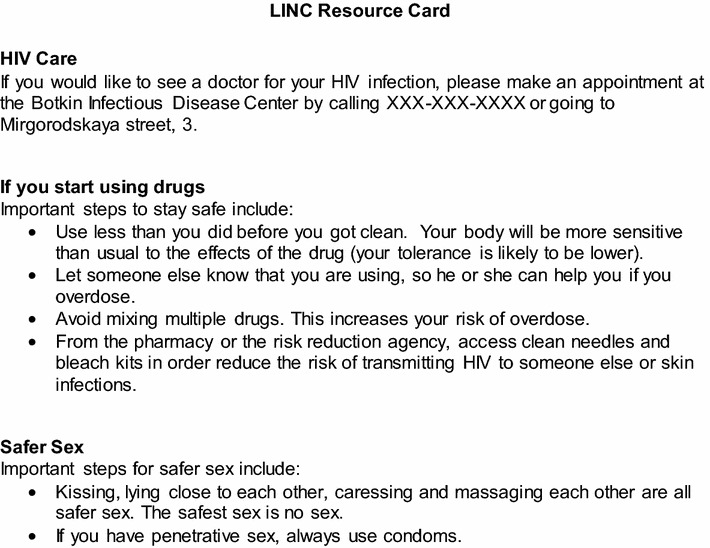
LINC resource card containing harm reduction information and contact information for the local HIV clinic

After enrollment into the study, the RAs asked participants if they were newly diagnosed with HIV and if so, whether or not they had received post-test counseling. The RA administered HIV post-test counseling to any newly diagnosed patients who had not yet received it. Over the course of the study, six patients were newly diagnosed and received post-test counseling from the study RA. The post-test counseling typically lasted 10 min and was conducted according to the guidelines of the Russian Ministry of Health, which were consistent with the US Centers for Disease Control and Prevention (CDC) recommendations. Following post-test counseling, if indicated, the RA collected locator information for the participant and alternate contacts. The RA then administered the baseline assessment and randomized the participant into the intervention or control group. The RA accompanied the participant to the laboratory for CD4 testing and provided the participant with a resource card containing harm reduction information and contact information for the local HIV clinic (Fig. 2).

### Randomization

Randomization was stratified on two factors: (1) whether an outpatient appointment with an infectious disease physician occurred in the 12 months prior to enrollment, and (2) whether the participant reported ever having been hospitalized for his or her HIV infection. Stratified randomization was used in order to ensure balance with respect to these potential confounding factors. Blocked randomization using random block sizes was used within each stratum. A computer-generated randomization table was created allowing randomization to occur via a custom web-application. Due to the nature of the intervention, participants and case managers could not be blinded to group assignment. However, the study was designed to minimize measurement bias by having the baseline assessment administered prior to randomization and by concealing randomization assignment from the follow-up assessors.

### Intervention

The LINC intervention was a behavioral and structural intervention designed to support and motivate HIV-infected PWID to engage in (i.e., initiate and retain) HIV medical care and ultimately improve their HIV outcomes. This intervention involved coordination between the narcology and HIV systems of care, utilizing HIV strengths-based case management delivered via five one-on-one sessions by a peer case manager (i.e., HIV-infected men and women in recovery from addiction) to help motivate and reduce barriers to HIV care. The initial session was designed to be delivered in the narcology hospital and included provision of CD4 test results by the case manager (CM) in a timely fashion, to increase engagement in HIV medical care. If the participant departed the City Addiction Hospital prior to having their initial intervention session, then this encounter was pursued outside of the hospital. Subsequent sessions were conducted upon discharge from the narcology hospital over a 6-month period in community (e.g., parks, coffee shops) or clinic locations, agreed upon by the case manager and participant.

The LINC intervention was developed via adaptation of the Antiretroviral Treatment Access Study (ARTAS) intervention for use in the Russian setting and specifically with PWID [22]. The ARTAS model was developed in the United States for a spectrum of HIV-infected persons and not specifically for PWID. Consistent with the ARTAS model, the LINC intervention used a strengths-based case management approach in which a trained CM met individually with clients to motivate them to engage in HIV medical care by supporting the recognition of their own strengths to make positive changes in their lives. This approach was grounded in a Social Cognitive Theory (SCT) and Psychological Empowerment Theory (PET) framework [23, 24].

SCT posits that behavioral goals (e.g., HIV care initiation and maintenance) are achieved when individuals (1) perceive their capacity to engage in the behavior (self-efficacy), (2) have the skills to manage “triggers,” such as substance use that can impede the behavior (self-management) and (3) believe the behavior will be both beneficial and socially supported (outcome expectancies) [23]. Additionally, SCT recognizes that such factors can only impact behavior, such as engagement in HIV care, in an environment where access to services exists [25]. Hence, the LINC intervention offered strengths-based case management in the addiction treatment setting and in transition to the HIV care setting in order to (1) build self-efficacy, (2) enable self-management, and (3) increase outcome expectancies regarding engaging in HIV care via education and social support.

Use of SCT is predicated on individuals having some degree of personal empowerment to create change in their own lives. Given the marginalization of HIV-infected PWID in Russia, this approach benefits from use of PET to bolster the effectiveness of the SCT-derived intervention [24]. According to PET, empowerment, the process by which individuals gain power over their own lives, occurs with opportunities to (1) influence the system (e.g., HIV clinical care) affecting them (intrapersonal), (2) have control over their role in transactions (interactional), and (3) contribute to changing the system and its interactions with them to better meet their health needs respectfully (behavioral). The LINC strengths-based HIV case management was designed to promote patient empowerment by providing education on how to control the HIV and addiction care they receive (intrapersonal), their right to receive and how to advocate for quality HIV and addiction care (interactional), and peer HIV case management by HIV-infected PWID in recovery to model for patients how to pursue healthy and productive lives (behavioral).

Six peer case managers (CMs), who were HIV-infected and in recovery (i.e., history of substance use disorder), were hired and trained to conduct the LINC intervention. All CMs had experience providing case management to substance using and/or HIV-infected adults in St. Petersburg. The LINC intervention was developed, and training was organized and led by the team of US and Russian investigators. Researchers (AR, JS, EB) were trained by Richard Rapp, MSW in a two-day training in Boston, which focused on the Antiretroviral Treatment Access Study (ARTAS) Linkage Case Management (ALCM) method [22]. Over the course of 3 days, researchers (AR, JS, EB) trained the CMs in St. Petersburg, providing them with an overview of the theoretical framework, assessment techniques and the LINC intervention. Researchers modeled and role-played the intervention delivery for the CMs to assure the use of a strengths-based approach. A simultaneous interpreter was used to allow the role-playing sessions to be critiqued. The team covered a number of topics with the CMs: HIV in PWID populations, addiction treatment options, HIV care, and antiretroviral therapy adherence. Booster trainings were conducted annually and as necessary, based on findings from quality assurance efforts. These intensive trainings, as well as the monitoring and observation for quality assurance were designed to limit potential variability due to individual CMs.

Each participant randomized to the intervention was assigned a specific CM, to ensure that the participants would be distributed evenly among the CMs. Upon randomization to the intervention, the RA notified the assigned CM via text message or phone call to meet with the participant at the City Addiction Hospital within 3 days. Reassignment to an alternate CM did occasionally occur due to CM illness, but efforts were made to maintain the same CM for each participant for the duration of the intervention.

The first case management session was held at the City Addiction Hospital with sessions 2–5 occurring anywhere in the community, or via phone if necessary. A session could also occur in the HIV clinic, to facilitate HIV care acquisition. The first session consisted of ascertaining the client’s strengths and developing goals related to obtaining HIV care, as well as discussing with the client their most recent CD4 cell count and benefits of HIV care (see Table 2 for a complete list of first session components). Follow-up sessions were based on a similar curriculum to the first session, but were intended to be shorter and more focused on meeting previously set goals and creating new goals based on the client’s previously self-identified strengths. Emphasis was placed on CMs guiding the client towards receiving HIV care without coercion, and supporting the client’s strengths and capacities to achieve his/her goals.

**Table 2:** LINC 1ST intervention session components

Brainstorming client strengths
Developing goals related to obtaining HIV and addiction care
Showing an informational video about HIV and ART
Providing a map of location of HIV clinics
Providing phone numbers to the HIV clinic
Discussing barriers to receiving HIV care and how to overcome them
Discussing what case management was and how the case manager could assist the client
Discussing options for addiction care
Discussing the client’s most recent CD4 cell count and what it means in terms of HIV care and receiving ART

The CM contacted participants between case management sessions by phone, text message, and/or email to remind them of upcoming appointments and to check-in. CMs used their clinical judgment to determine how frequently to provide check-in calls. At a minimum, a reminder text message and/or email using a standard script was sent to participants within a week prior to their next case management session. CMs maintained detailed clinical notes and contact logs, as well as brief tracking forms to record the location and duration of sessions and check-ins.

### Control condition

Participants randomized to the control condition received the narcology hospital’s standard of care. With regard to linkage to HIV medical care, RAs provided all participants with a resource card containing harm reduction information and contact information for the local HIV clinic (Fig. 2).

### Implementation assessments

Several implementation assessments were utilized to determine the adherence to and the fidelity of the LINC intervention.

*Case Manager Checklists* were completed by the CMs during all case management sessions. These checklists served to identify the content for the intervention, providing an outline of the CM activities and discussion points.

*Case Manager Observations* were conducted by the Russian project manager via audiotaped recordings of the first three sessions for each CM and 10 % of all subsequent sessions. A checklist was completed by the Russian project manager to document the level of CM adherence to the content and participant engagement.

*Clinical Supervision and Feedback* was an integral component of the process evaluation strategy. The Russian project manager, a trained physician narcologist, reviewed CM clinical notes, contact logs, CM checklists and observation forms, and met with CMs monthly to provide feedback and conduct any additional training as needed. Feedback was provided using a strengths-based supervision approach. The Russian project manager met regularly with the research team’s behavioral psychologist (AR) and the US project manager to discuss any challenges that were raised during the clinical supervision meetings with CMs and throughout the course of the intervention.

*Case Manager Evaluation Forms* assessed CM satisfaction with the LINC intervention. Every 3 months after the start of the LINC intervention, the CMs provided their opinions on the effectiveness of the intervention and whether or not it helped participants link to HIV care. The evaluation also asked CMs about any difficulties they encountered during the intervention.

*Participant Satisfaction Survey* was a self-administered survey completed by study participants at the time of their 6-month study research assessment. It assessed participant satisfaction with case management sessions and whether CMs helped participants with any health issues related to HIV infection or addiction.

### Research assessments

Study interviews were conducted face-to-face by trained research staff at baseline (pre-randomization) at the narcology hospital, and at 6 and 12-months post-enrollment at First St. Petersburg Pavlov State Medical University. In the event of readmission, follow-up research interviews were occasionally conducted at the City Addiction Hospital. Participants were compensated for completion of each study visit (Table 3).

**Table 3:** Study activities and assessment

Study activity	Baseline LINC	6-Month follow up LINC	12-Month follow up LINC
Study Assessment	x	x	x
Blood collection for CD4 cell count	x		x
Medical chart reviews^a^		x	x
Payments	$10	$50	$50

^a^Also performed during pre-screening

The components of the baseline and follow-up assessments and study activities are shown in Tables 3, 4 and 5. Assessment components were selected to maximize harmonization efforts with other investigators supported to pursue research projects in the National Institute on Drug Abuse (NIDA) STTR portfolio [26, 27]. Most sections of the assessment were interviewer-administered, while sections containing potentially sensitive questions (e.g., questions regarding sexual partners, HIV disclosure, HIV stigma, depressive symptoms, partner violence and sexual assault) were self-administered. All assessment data were entered directly into study computers. During a reminder call that took place 3 months post-enrollment, participants were asked about the number of times they had overdosed since the baseline interview.

**Table 4:** Components of LINC baseline assessment

Baseline assessment components	Description
Demographics [31]	Participant demographics and socio-economic status
HIV Testing and HCV Diagnosis [32]	Dates and locations of HIV/HCV testing and HIV/HCV treatment
Health Care Utilization	How often participants see a physician and if they receive treatment for HIV and addiction
Access to Care Scale [33, 34]	Participant perceptions of access to medical care
Barriers to Medical Care [35]	Reasons why participants may not have received medical care in the past month
Health Literacy [36]	Participant understanding of medical information and forms
HIV Symptom Index [37]	A 20-item HIV symptom index of patient-reported symptoms
Sexually Transmitted Diseases	Assesses if participants have ever been treated for sexually transmitted diseases
HIV Sex Risk Behaviors [38]	Questions about vaginal, oral, and/or anal sexual practices with any sexual partners and use of substances before or during sex
Sexual Partners^a^	Questions about number and gender of recent sexual partners
HIV Disclosure^a^ [39, 40]	Discussion of HIV status with others, including sexual partners
HIV Stigma^a^ [41]	Experiences and feelings participants may have because of HIV
Center for Epidemiologic Studies-Depression Scale (CES-D)^a^ [42, 43]	A measure of depressive symptomatology
State Trait Anxiety Inventory (STAI)– Short Form^a^ [44, 45]	A measure of current feelings of anxiety
Partner Violence and Sexual Assault^a^	Experiences of having been hurt physically or sexually
The Fagerstrom Test for Nicotine Dependence [46, 47]	A measure of nicotine dependence
Alcohol Use Disorders Identification Test (AUDIT) [48]	Identifies persons with hazardous alcohol use
Texas Christian University (TCU): Drug Screen [49, 50]	Assesses drug use in the past year
Drug Use [51, 52]	Assesses frequency and type of drugs used and drug risk behaviors
Overdose and Suicide [53]	Questions regarding overdose and suicide attempts
Household Food Insecurity Access Scale (HFIAS) [54]	Assesses access to food in the past 4 weeks
Barratt Impulsiveness Scale [55]	Assesses participant impulsivity
Social Support Scale [56]	Measures access to companionship, assistance, or other types of support
Involvement with Police [57]	Assesses experiences with police officers
RAND36 Health Survey [58]	General assessment of overall health

A number of measures used were modified from validated instruments

^a^Self-administered sections

**Table 5:** Components of the LINC 6- and 12-month assessment

Follow up assessment components^a^	Description
ART Use and Adherence [59–61]	Questions on ART use and 30-day adherence
Opportunistic Infections [62]	Assesses any history of candida or yeast infection of the esophagus, TB, pneumonia, or toxoplasmosis
Barriers to Medical Care: Drug Use [63]	Assesses if drug use has affected access to medical care
Perceived Discrimination in Health Care^b^ [64]	Assesses participant’s perceived discrimination in health care because of their drug use
Case Manager Questions^b^	Assess participant’s feelings and experiences related to case management
Reproductive Health [65]	Questions regarding reproductive health for female participants

A number of measures used were modified from validated instruments

^a^Also includes all Baseline Assessment Components except Health Literacy, Sexually Transmitted Diseases, HIV Disclosure, and Involvement with Police; 12-month Assessment Components are the same as 6-month Assessment Components with the exception of Reproductive Health section (6-months only)

^b^Self-administered section

In addition to study questionnaires, at each study visit RAs measured and recorded participants’ blood pressure, height, and weight. Blood was collected for CD4 count testing at baseline and 12-months.

### Outcomes

**Fig. 3:**
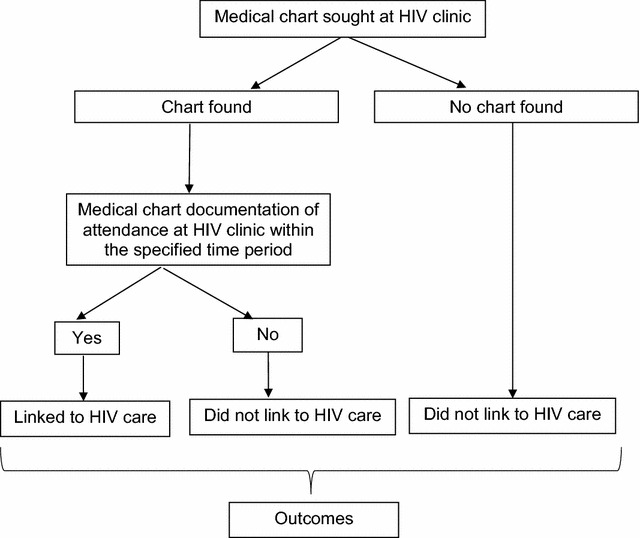
LINC medical record review flow chart

The study aimed to test the hypotheses that participants randomized to the LINC intervention would have better: (1) initiation of HIV care; (2) retention in HIV care; (3) receipt of appropriate HIV care; and (4) HIV health outcomes, as compared to the control group. The primary outcome for hypothesis 1 (initiation of HIV care) was defined as at least one HIV physician appointment within 6 months of enrollment, assessed by medical record review (Fig. 3). A secondary outcome was self-reported initiation of HIV care. The primary outcome for hypothesis 2 (retention in HIV care) was defined as at least one HIV physician appointment in each of the two consecutive 6 month periods within 12 months of enrollment, assessed by medical record review. A secondary outcome was self-reported retention in HIV care. The primary outcome for hypothesis 3 was receipt of appropriate HIV care, defined as a CD4 cell count assessed and ART prescribed if CD4 is <350, or if >350 then another CD4 cell count obtained within 12 months. The primary outcome for hypothesis 4 was CD4 count obtained during the 12-month research visit. A secondary outcome was any self-reported hospitalizations.

Data were collected from patient medical charts to assess the study’s primary outcomes of participant initiation of and retention in HIV care, as well as the appropriateness of HIV care. Chart reviews were conducted at two central HIV care centers, where study participants were expected to seek HIV medical care: Botkin Infectious Disease Hospital and City AIDS Center. In Russia, government-funded care is sought and provided based on the patient’s address. Patients who seek addiction care at the City Addiction Hospital, the LINC recruitment site, would receive HIV care at either the City AIDS Center or Botkin Infectious Disease Hospital. Although eligibility criteria stipulated that participants should be willing to receive care from Botkin Infectious Disease Hospital, they were not required to do so. After initial care is established at one of these main hospitals, patients are able to receive follow-up care at local clinics. At Botkin Hospital, every 3–4 months a study staff member conducted chart reviews on all participants, who were 6 or 12 months post-baseline, and entered data directly into a web-based chart review form. At the City AIDS Center chart reviews were conducted annually for all participants who were 12 months post-baseline by local hospital staff trained by a Russian co-investigator (EB) and the study staff member who conducted the chart review at Botkin Hospital. Chart review information was entered onto paper forms and subsequently transferred to the main research site at First St. Petersburg Pavlov State Medical University and entered into the online chart review form by a study staff member. For a small number (n < 20) of participants, whose City AIDS Center charts indicated the receipt of HIV care from a district clinic, or if participants reported receiving HIV care elsewhere, medical charts were sought for review from those locations. Data obtained from patient medical charts included clinic visit dates, CD4 testing dates and results, and ART information.

Loss to follow-up was a concern in this difficult-to-track population, which included not only those difficult to contact (e.g., actively using substances), but also deaths and incarcerations. However, loss to follow-up was minimized by using medical record review to obtain data for three of the four primary outcomes. The research team had previous experience with long-term follow-up of similar participants, analysis of incomplete longitudinal data, and medical record review of clinical data using a standard protocol, which minimized incomplete observations.

### Analytic methods

Descriptive statistics will be calculated for all variables at baseline, 6-, and 12-months. All baseline variables will be assessed to ascertain important differences across treatment arms. Spearman correlation analyses will be performed to identify pairs of covariates that are correlated (r > 0.4) and would therefore not be included together in regression analyses. All analyses will use the intention-to-treat approach and analyze participants according to randomized group.

To assess initiation, retention and appropriateness of HIV medical care, initial analyses will be performed comparing these binary outcomes between groups using a Chi square test. The primary analysis will use multivariable logistic regression analyses to control for stratification factors (i.e., outpatient appointment with an HIV doctor in the past 12 months, ever having been hospitalized for HIV infection) in order to improve efficiency. Exploratory analyses will be conducted to examine whether or not there were effects due to HIV care site. The primary outcome for HIV health (i.e., CD4 cell count at 12 months) is continuous and will be analyzed using multiple linear regression models controlling for stratification factors and any baseline characteristics that differ.

Power calculations assumed a 2-sided test, with a significance level of 0.05. It was expected that 350 patients would be enrolled into the study. Because the primary outcome for the overall study, initiation of HIV care, was assessed using medical records, we expected loss to follow-up to be minimal. Based on a prior study from this research team (i.e., the HERMITAGE study), we estimated a 10 % loss to follow-up due to death and participant withdrawals [28]. According to data collected at another Russian narcology hospital, the Leningrad Regional Center for Addiction, by our Russian collaborators (EK, EB), we expected 20 % of controls to attend one or more appointments in 6 months. Based on these assumptions and with 315 evaluable participants, the study has 80 % power to detect an absolute difference of 15 % (i.e., 35 vs. 20 % in the intervention and control groups, respectively) in the proportions initiating HIV care using a Chi square test with continuity correction.

### Protection of study participants and study data

The LINC study was approved by the Institutional Review Boards of Boston University Medical Campus and First St. Petersburg Pavlov State Medical University. All study participants completed the informed consent process and provided written informed consent. Serious Adverse Events (i.e., hospitalizations and deaths) were reported by the study team to the study Principal Investigator (JS), as the team became aware of their occurrence. Events were reported to the Institutional Review Board annually.

With the exception of medical record review at the City AIDS Center, which was collected on paper and entered later, all study data were captured electronically on study computers via a secure, web-based data capture system. Access to the system was protected via secure logins and all data transmissions were encrypted using secure socket layering. Study data were housed on secure servers maintained by the Boston University Office of Information Technology, behind the Boston University firewalls. Identifiers needed to track participants were kept separate from research data in a secure database to which access was restricted.

## Discussion and potential impact

The LINC study tests the effectiveness of a pragmatic intervention to engage HIV-infected PWID in HIV medical care by attempting to link HIV and narcology systems within the Russian medical care context. Given that a substantial fraction of Russian HIV-infected PWID are untreated in terms of their HIV infection, and current HIV testing for all patients at high risk for HIV already occurs as standard practice in narcology hospitals, an effective linkage clinical approach would be of great value [29]. Adapting an approach demonstrated to improve linkage in the United States is a reasonable start, but much is different in the Russian context and hence extensive efforts to adapt and train the “peer” case managers, as described in this protocol, are necessary. Since opioid agonist treatment (OAT) is not permitted nor accessible in Russia, the LINC intervention would be carried out primarily among people *actively* injecting drugs, typically opioids. According to Russian guidelines ART is initiated at CD4 cell counts below 350 cells/mm3; these guidelines are not conditional on one’s drug use behavior. The pragmatic features of the LINC intervention are important, as hospitals could utilize the relatively new, but already existing position of HIV case managers (CMs) in some parts of Russia. Thus, the LINC protocol could be implemented without significantly changing the structure of care delivery in Russia. The peer-led case management sessions being tested in this LINC study empower narcology hospital patients to achieve goals that they set for themselves and work to keep the patients accountable for these goals throughout the case management sessions.

This LINC study will examine whether the intervention will improve the initiation and retention of participants in HIV care, lead to receipt of appropriate HIV care and result in better HIV health outcomes. It is clear that the intervention most directly targets the process of initiation of care and will depend on a functioning HIV health care system to facilitate subsequent health outcomes. This is a logical consequence demonstrated in other settings, in which a higher percentage of HIV patients who received more frequent and continuous HIV care achieved viral suppression, compared to those who received HIV care less frequently, but its occurrence in the Russian setting merits examination as per the study protocol [30]. The LINC intervention optimizes resources already available in Russia to initiate and retain HIV-infected PWID in appropriate HIV care. It will potentially address a critical aspect of the seek, test, treat and retain (STTR) strategy to contribute to the global effort to improve health systems’ performance to advance HIV-infected individuals down the HIV care cascade, particularly PWID within the addiction treatment setting.

## Abbreviations

ART: antiretroviral therapy
ARTAS: Antiretroviral Treatment Access Study
CM: case manager
LINC: Linking Infectious and Narcology Care
PET: Psychological Empowerment Theory
PWID: people who inject drugs
RA: Research Assessor
SCT: Social Cognitive Theory
STTR: seek, test, treat and retain
